# Detection and prognostic role of circulating cancer-associated fibroblasts in the blood of melanoma patients

**DOI:** 10.3389/fcell.2026.1774206

**Published:** 2026-06-01

**Authors:** Kim-Lea Reese Ryterski, Hyeong Jung Woo, Svenja Schneegans, Lina Bergmann, Ann-Kristin Afflerbach, Mathieu Garcia, Noah Zimmermann, Isabel Heidrich, Glenn Geidel, Julian Kött, Stefan W. Schneider, Christoffer Gebhardt, Minseok S. Kim, Klaus Pantel, Daniel J. Smit

**Affiliations:** 1 Institute of Tumor Biology, University Medical Center Hamburg-Eppendorf, Hamburg, Germany; 2 Department of New Biology, Daegu Gyeongbuk Institute of Science & Technology (DGIST), Daegu, Republic of Korea; 3 Bioinformatics Core, University Medical Center Hamburg-Eppendorf, Hamburg, Germany; 4 Department of Dermatology and Venereology, University Medical Center Hamburg-Eppendorf, Hamburg, Germany; 5 Fleur Hiege Center for Skin Cancer Research, University Medical Center Hamburg-Eppendorf, Hamburg, Germany; 6 CTCELLS Inc., Daegu, Republic of Korea

**Keywords:** cCAF, circulating cancer-associated fibroblasts, circulating tumor cells, liquid biopsy, melanoma

## Abstract

**Background:**

Cancer-associated fibroblasts (CAFs), frequently present in many tumor tissues, have received increasing attention over the past decade, while research on CAFs circulating in the blood of cancer patients is still in its infancy. This is the first study to assess the incidence, concentration, and potential prognostic value of cCAFs alone or in combination with other biomarkers such as circulating tumor cells (CTCs) or cancer-associated proteins, in melanoma patients.

**Methods:**

CTCs and cCAFs were enriched from whole blood samples of 31 melanoma patients (stage IIB–IV) using the CTCeptor system, which makes use of automated density-based enrichment and CD45-based negative depletion. The isolated cells were stained with DAPI, and antibodies against MART-1, MCAM, α-SMA, and CD45. CTCs were defined as DAPI+, MART-1/MCAM+, CD45^−^cells, while cCAFs were defined as DAPI+, α-SMA+, CD45^−^cells.

**Results:**

CTCs and cCAFs were detected in approximately half of the melanoma patients, respectively. On average, more cCAFs (mean: 11 cells, range: 1–60) than CTCs (mean: 4.5 cells, range: 1–20) were found in the patients’ blood samples. The median progression-free survival (PFS) for patients with an increased cCAF count (≥5) was 2.07 months, while for those with a lower cCAF count (<5), it was 10.35 months (p = 0.51). When combined with elevated lactate dehydrogenase (LDH) (≥245 U/L) or S100B (≥0.152 μg/L) levels, high cCAF counts tend to a reduced PFS (high LDH/high cCAF: 1.92 months, high S100B/high cCAF: 1.77 months), compared to patients with low LDH/S100B, indicating improved risk stratification when cCAFs are used alongside established biomarkers.

**Conclusion:**

This study demonstrates the possibility of co-detecting CTCs and cCAFs in the blood of melanoma patients for the first time. A higher mean number of cCAFs was detected and showed a trend toward shorter progression-free survival. The encouraging results of this pilot study need to be validated on a larger cohort of melanoma patients.

## 1 Introduction

Malignant melanoma stands out among different types of skin cancer due to its aggressive nature and the rising numbers of new cases and deaths over the past decades (Tímár and Ladányi, 2022). Despite advances in therapies, particularly immune checkpoint inhibitors (ICIs), including anti-CTLA-4 and anti-PD-1 antibodies, not all patients achieve a durable response, resulting in many experiencing disease progression (Larkin et al., 2019; Noringriis et al., 2025). Thus, the discovery of novel biomarkers to identify these patients and to offer personalized therapy is urgently required.

The tumor microenvironment (TME) plays a crucial role in the response to immunotherapies, as it comprises immune cells, stromal cells, extracellular matrix, and secreted molecules (Turner et al., 2025). Specifically, cancer-associated fibroblasts (CAFs) have been shown to influence the TME by secreting or directly transporting various cytokines, including TGF-β, IL-6, and IL-10, to other cells, and by creating physical barriers through remodeling of the extracellular matrix (Gaggioli et al., 2007; Sahai et al., 2020). Typically, CAFs are associated with reduced T-cell infiltration and poorer therapeutic responses; however, some CAF subtypes also exhibit anti-tumor properties (Raaijmakers et al., 2024; Zheng et al., 2025). So far, the functional role of CAFs as biomarkers remains unclear, and their analysis depends on the availability of suitable tissue samples.

Liquid biopsy enables the minimally invasive and repeatable analysis of circulating tumor-derived components in several body fluids, such as blood (Alix-Panabières and Pantel, 2021; 2025a; b; Pantel and Alix-Panabières, 2025). While other rare cells in the blood, like circulating tumor cells (CTCs), have been studied extensively in solid tumors and also melanoma (Felici et al., 2022; Shoji et al., 2022; Sementsov et al., 2024), circulating CAFs (cCAFs) have been identified and characterized in only a limited number of studies, but not yet in melanoma (Jones et al., 2013; Ao et al., 2015; Ortiz-Otero et al., 2020; Jiang et al., 2021; Sharma et al., 2021; Muchlińska et al., 2023; Booijink et al., 2024; Götze et al., 2025). In some studies, patients with metastasis showed increased numbers of cCAFs compared to patients with localized tumors, including breast, lung, and gastrointestinal cancers (Weber et al., 2025). cCAFs have been found in heterotypic clusters with other cells, such as CTCs, which exhibit a higher metastatic potential than homotypic clusters composed only of tumor cells (Hurtado et al., 2020; Sharma et al., 2021). It is hypothesized that cCAFs could contribute to tumor dissemination by protecting tumor cells in circulation, facilitating their survival, or helping to establish a supportive microenvironment at distant, pre-metastatic sites (Szczerba et al., 2019; Hurtado et al., 2020; Eslami et al., 2023). However, the functional role and clinical relevance of cCAFs are only partially understood, and no data exist for melanoma yet.

Since reliable prognostic biomarkers for monitoring treatment response and guiding therapy decisions in melanoma are lacking, this study aimed to examine cCAFs and CTCs as potential new biomarkers. To our best knowledge, this is the first study to isolate cCAFs from the blood of melanoma patients and analyze their clinical association with demographic, clinico-pathological, and survival data during immunotherapy.

## 2 Materials and methods

### 2.1 Study cohort and blood sample collection

Melanoma patients were prospectively recruited at the Department of Dermatology and Venereology, University Skin Cancer Center of the University Medical Center Hamburg-Eppendorf between January 2024 and May 2025. In total, 31 treatment-naïve melanoma patients were recruited. Clinico-pathological data were obtained from records at the University Medical Center Hamburg. The tumor stage was encoded according to the 8^th^ edition of the melanoma staging system by the American Joint Committee on Cancer (AJCC) (Keung and Gershenwald, 2018). The cut-off values for lactate dehydrogenase (LDH) and S100B were chosen based on established clinical reference ranges. Peripheral blood samples were collected into EDTA tubes (S-Monovette Potassium-EDTA, Sarstedt) and processed within 4 h after collection. The study was approved by the Ethics Committee of the Chamber of Physicians Hamburg, Germany, under permit number PV5392. Written informed consent was obtained from all patients.

### 2.2 Enrichment of CTCs and cCAFs

Cells of interest were enriched from whole blood using the CTCeptor system (CTCELLS Inc.) as described previously (Woo et al., 2022; Kang et al., 2023). In brief, density gradient media (Ficoll Premium, Cytiva) and 3 mL of whole blood were layered and centrifuged to separate peripheral blood mononuclear cells (PBMCs) from red blood cells and plasma. The isolated PBMCs were incubated with 75 µL of anti-CD45 conjugated magnetic beads (Dynabeads CD45, Invitrogen), layered on 875 µL of density gradient media, and centrifuged to deplete white blood cells and enrich the CTCs and cCAFs. The retrieved cells were washed in PBS and centrifuged (176 x *g*, 7 min) on glass slides. After drying, the slides were stored at −80 °C in aluminum foil until immunocytochemistry (ICC) staining.

### 2.3 Immunocytochemical staining of enriched cells

Previously prepared cytospins were thawed at room temperature, and the isolated cells were fixed with 0.5% paraformaldehyde (#28908, ThermoFisher Scientific) at room temperature for 10 min. Thereafter, the cells were washed three times with PBS for 3 minutes each, and 0.5% Triton-X100 (#9002-93–1, Sigma Aldrich) was added for permeabilization for 3 minutes, followed by another wash using PBS. A 2% bovine serum albumin (BSA) (#A7030-100G, Sigma Aldrich) solution was used to block nonspecific binding before incubating the cells with the following antibodies in 2% BSA overnight at 4 °C: anti-CD45 (#304018, Biolegend, 1:400), anti-α-SMA (#PA5-18292, Invitrogen, 1:400), anti-MART-1 (#64718S, Cell Signaling Technology, 1:200), anti-MCAM (#81701S, Cell Signaling Technology, 1:200), and DAPI (#6335.1, Carl Roth, 1:1000). After washing with PBS, appropriate secondary antibodies Alexa Fluor 546 donkey anti-goat (#A11056, Invitrogen, 1:500) and Alexa Fluor 488 donkey anti-rabbit (#A21206, Invitrogen, 1:1000) were added at room temperature for 1 hour. The slides were washed with PBS, then the entire slide was imaged using the Axio Observer 7 (Zeiss) fluorescence microscope at ×10 magnification. Cells with intact morphology that were positive for MART-1/MCAM and DAPI, but negative for CD45 and α-SMA, were classified as CTCs, and cells positive for α-SMA and DAPI, but negative for CD45 and MART-1/MCAM, were classified as cCAFs. At least two independent researchers analyzed all slides.

### 2.4 Statistical analysis

Statistical analysis was conducted using R (version 4.5.1) in the RStudio environment (version 2025.09.2 + 418) with the following packages: finalfit (version 1.0.8) (Harrison et al., 2021), survminer (version 0.5.0) (Kassambara and Biecek, 2021), and tidyverse (version 2.0.0) (Wickham, 2023). Laboratory measurements of CRP that were below the detection limit were set to half the lower limit for further analysis. Categorical data were evaluated using Fisher’s exact test. Continuous data were tested for normal distribution using the Shapiro-Wilk test. Non-parametric continuous variables were analyzed by the Mann-Whitney U test and reported as median with interquartile range (IQR). Parametric continuous variables were evaluated for variance equality using the Levene test. In case of homoscedasticity, mean differences were analyzed by a two-tailed Student’s t-test. The optimal cut-off for CTCs and cCAFs was calculated using maximally selected rank statistics based on overall survival. Survival data were plotted using the Kaplan-Meier method, and statistical significance was analyzed with the log-rank test. For plots with more than two groups, the reported p-values of the log-rank test were adjusted for multiple testing by the Holm-Bonferroni method. All statistical tests were conducted as two-sided tests. A p-value lower than 0.05 was considered statistically significant.

## 3 Results

### 3.1 Clinico-pathological characteristics of melanoma patients

In our study, we prospectively included 31 patients with histologically confirmed melanoma who were treatment-naïve for immune checkpoint inhibitor therapy (Table 1). 27 patients presented with cutaneous melanoma, three with uveal melanoma, and one with melanoma of unknown primary (MUP). The average age of the patients was 68.2 years. Of the 31 patients, 18 were female (58.1%), and 13 were male (41.9%). Most patients had distant metastasis and were classified as AJCC stage IV (n = 19), while the remaining distant metastasis-free patients were classified as AJCC stage III (n = 11) and one as AJCC stage IIB. PD-1 inhibitor monotherapy was administered to 14 patients, 14 patients received combination therapy with CTLA-4 and PD-1 inhibitors, and three patients did not receive therapy. As the best overall response, one patient had complete remission, seven showed no evidence of disease, one had partial remission, four had stable disease, and eight had progressive disease at the time of database lock.

**TABLE 1:** Descriptive statistics of the demographic and clinico-pathological parameters of the study cohort**.**

Variable	Total n [%]	Level	n [%/SD]
Age	31 (100.0)	Mean (SD)	68.2 (12.9)
Sex	31 (100.0)	Female	18 (58.1)
​	​	Male	13 (41.9)
Primary melanoma site	31 (100.0)	Cutaneous	27 (87.1)
​	​	Uveal	3 (9.7)
​	​	MUP	1 (3.2)
AJCC stage	31 (100.0)	IIB	1 (3.2)
​	​	IIIB	6 (19.4)
​	​	IIIC	4 (12.9)
​	​	IIID	1 (3.2)
​	​	IV	19 (61.3)
T	31 (100.0)	T0	1 (3.2)
​	​	T1	4 (12.9)
​	​	T2	3 (9.7)
​	​	T3	7 (22.6)
​	​	T4	10 (32.3)
​	​	Tx	6 (19.4)
N	31 (100.0)	N0	11 (35.5)
​	​	N1	10 (32.3)
​	​	N2	5 (16.1)
​	​	N3	5 (16.1)
M	31 (100.0)	M0	12 (38.7)
​	​	M1	3 (9.7)
​	​	M1a	3 (9.7)
​	​	M1b	1 (3.2)
​	​	M1c	6 (19.4)
​	​	M1d	6 (19.4)
Therapy	28 (90.3)*	Anti-CTLA-4 + anti-PD-1	14 (50.0)
​	​	Anti-PD-1	14 (50.0)
Best overall response	21 (67.7)	Complete remission	1 (4.8)
​	​	No evidence of disease	7 (33.3)
​	​	Progressive disease	8 (38.1)
​	​	Partial remission	1 (4.8)
​	​	Stable disease	4 (19.0)

AJCC, american joint committee on cancer; T, tumor size (Tx, primary tumor cannot be assessed); N, lymph node positivity; M, distant metastasis; MUP, melanoma of unknown primary; PD-1, programmed cell death protein 1; CTLA-4, cytotoxic T-lymphocyte-associated protein 4.

* Three patients did not receive therapy.

### 3.2 CTCs and cCAFs are present in the blood of melanoma patients

**FIGURE 1:**
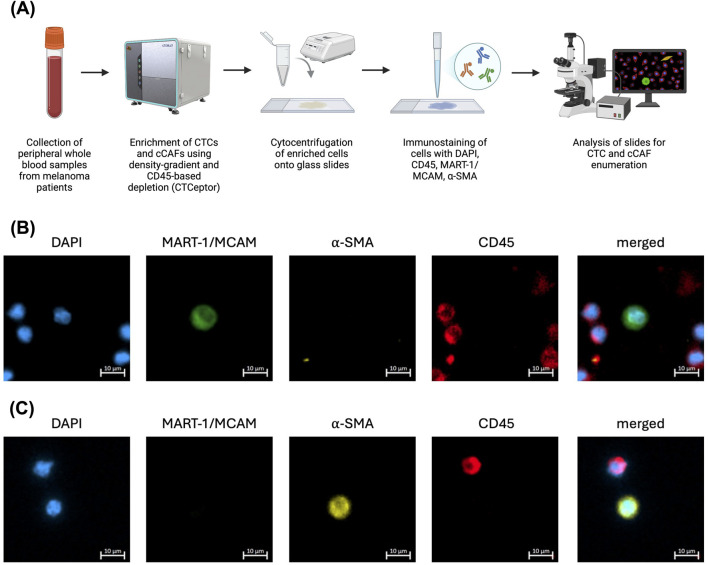
Enrichment and characterization of CTCs and cCAFs. **(A)** Graphical overview of the methodology to enrich and detect CTCs and cCAFs from whole blood samples of melanoma patients. Created in BioRender. Reese K. (2026) https://BioRender.com/az96xj6. Representative fluorescence images of **(B)** CTCs and **(C)** cCAFs were taken with 10x magnification. The scale bar represents 10 μm.

We processed whole blood samples using the CTCeptor system, based on automated density-gradient and CD45-based depletion to detect CTCs and cCAFs in the blood of melanoma patients (Figure 1A). An immunocytochemical staining protocol was developed to co-detect CTCs and cCAFs from the same patient sample. CTCs were defined as DAPI^+^, MART-1/MCAM^+^, α-SMA^-^, and CD45^−^ cells, while cCAFs were identified as DAPI^+^, MART-1/MCAM^−^, α-SMA^+^, and CD45^−^ cells. Representative immunocytochemical staining images for CTCs and cCAFs are shown in Figures 1B,C, respectively.

**FIGURE 2:**
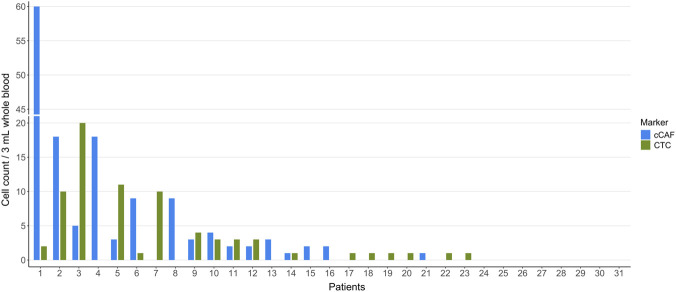
Histogram of cCAFs and CTCs found in 3 mL of whole blood for each patient. In our cohort, cCAFs (blue bar) were detected in 16 patients, and CTCs (green bar) were detected in 17 patients. Ten patients were positive for both cCAFs and CTCs, and eight patients had no detectable CTCs or cCAFs in their blood samples.

CTCs were detected in 17 of 31 (54.8%) patients with a mean of 4.5 CTCs, ranging from 1 to 20 CTCs. The percentage of patients positive for CTCs was higher in those with distant metastasis (63.2%; 12 out of 19) compared to those with locally (advanced) tumors (41.7%; 5 out of 12), but this difference was not statistically significant (p = 0.288). cCAFs were detected in 16 patients (51.6%) with a higher mean number of 11 cCAFs (range: 1–60) compared to CTCs. Among patients with distant metastasis, 52.6% (10 out of 19) had detectable cCAFs, similar to the cCAF positivity rate of 50% (6 out of 12) in patients without distant metastasis. Ten patients had both detectable CTCs and cCAFs, whereas eight patients had no CTCs or cCAFs (Figure 2). Neither cCAF positivity nor absolute counts correlated with CTC positivity or counts (Fisher’s exact test: p = 0.6638, Spearman ρ = 0.12, p = 0.733, respectively).

We evaluated whether CTC and cCAF counts were associated with the demographic, clinical, and pathological parameters before treatment initiation. As standardized thresholds or cut-off values for neither CTCs nor cCAFs in melanoma exist, we established the optimal cut-off for prognostic power using the maximally selected log-rank statistic based on overall survival. For CTCs, the optimal cut-off was two CTCs, whereas for cCAFs, the optimal cut-off was five cCAFs. Based on these cut-off values, patients were dichotomized, and the CTC/cCAF status was associated with demographic, clinico-pathological, and laboratory parameters.

No statistically significant associations were found between the CTC groups with respect to their demographic data, including age and sex, as well as their clinico-pathological data, including primary melanoma site, AJCC stage, TNM, therapy regimen, and clinical outcomes (Table 2).

**TABLE 2:** Descriptive statistics of the demographic and clinico-pathological parameters of the study cohort according to the CTC groups.

Variable	Total n [%]	Level	CTC <2	CTC ≥2	p-value
Sex	31 (100.0)	Female	13 (59.1)	5 (55.6)	1.000 (a)
​	​	Male	9 (40.9)	4 (44.4)	​
Age	31 (100.0)	Mean (SD)	69.6 (11.3)	64.7 (16.2)	0.342 (b)
Primary melanoma site	31 (100.0)	Cutaneous	20 (90.9)	7 (77.8)	0.437 (a)
​	​	Uveal	1 (4.5)	2 (22.2)	​
​	​	MUP	1 (4.5)	0 (0.0)	​
AJCC stage	31 (100.0)	IIB	0 (0.0)	1 (11.1)	0.087 (a)
​	​	III	10 (45.5)	1 (11.1)	​
​	​	IV	12 (54.5)	7 (77.8)	​
T	31 (100.0)	T0	1 (4.5)	0 (0.0)	0.868 (a)
​	​	T1	3 (13.6)	1 (11.1)	​
​	​	T2	2 (9.1)	1 (11.1)	​
​	​	T3	5 (22.7)	2 (22.2)	​
​	​	T4	8 (36.4)	2 (22.2)	​
​	​	Tx	3 (13.6)	3 (33.3)	​
N	31 (100.0)	N0	5 (22.7)	6 (66.7)	0.137 (a)
​	​	N1	9 (40.9)	1 (11.1)	​
​	​	N2	4 (18.2)	1 (11.1)	​
​	​	N3	4 (18.2)	1 (11.1)	​
M	31 (100.0)	M0	10 (45.5)	2 (22.2)	0.418 (a)
​	​	M1	12 (54.5)	7 (77.8)	​
Therapy	28 (90.3)*	Anti-CTLA-4 + anti-PD-1	12 (57.1)	2 (28.6)	0.385 (a)
​	​	Anti-PD-1	9 (42.9)	5 (71.4)	​
Progressive disease	31 (100.0)	No	13 (59.1)	6 (66.7)	1.000 (a)
​	​	Yes	9 (40.9)	3 (33.3)	​
Death	31 (100.0)	No	18 (81.8)	6 (66.7)	0.384 (a)
​	​	Yes	4 (18.2)	3 (33.3)	​

AJCC, american joint committee on cancer; T, tumor size (Tx, primary tumor cannot be assessed); N, lymph node positivity; M, distant metastasis (according to TNM, classification); MUP, melanoma of unknown primary; PD-1, programmed cell death protein 1; CTLA-4, cytotoxic T-lymphocyte-associated protein 4; SD, standard deviation. (a) Fisher’s exact test; (b) two-sided T-test.

* Three patients did not receive therapy.

Patients with increased CTC numbers had significantly higher D-dimers (p = 0.042) and C-reactive protein (CRP) (p = 0.009) levels compared to patients with less than two CTCs, while LDH, S100B, as well as neutrophils and lymphocytes, and the neutrophil-lymphocyte-ratio (NLR) were not different between the two groups (Table 3).

**TABLE 3:** Laboratory characteristics of the patient cohort according to CTC groups**.**

Variable	Total n	Level	CTC <2	CTC ≥2	p-value
LDH [U/L]	31	Median (IQR)	252 (127.25)	369 (523)	0.177 (a)
S100B [µg/L]	30	Median (IQR)	0.22 (0.45)	0.31 (4.96)	0.533 (a)
D-dimers [mg/L]	26	Median (IQR)	0.64 (0.83)	1.77 (2.64)	**0.042** (a)
CRP [mg/L]	23	Median (IQR)	5 (17)	71.5 (110.5)	**0.009** (a)
Neutrophil count [x10^9^/L]	31	Median (IQR)	5.47 (1.75)	6.46 (1.96)	0.133 (a)
Lymphocyte count [x10^9^/L]	31	Mean (SD)	1.65 (0.58)	1.28 (0.51)	0.102 (b)
NLR	31	Median (IQR)	3.26 (2.4)	4.44 (4.86)	0.070 (a)

LDH, lactate dehydrogenase; CRP, C-reactive protein; NLR, neutrophil/lymphocyte ratio; SD, standard deviation; IQR, interquartile range. (a) Mann–Whitney U test; (b) Student’s t-test. Significant p-values are highlighted in bold font.

With respect to cCAF status, among the demographic and clinico-pathological variables, only a younger age (p = 0.014) was significantly associated with a higher cCAF status (Table 4).

**TABLE 4:** Descriptive statistics of the demographic and clinico-pathological parameters of the study cohort according to the cCAF groups.

Variable	Total n [%]	Level	cCAF <5	cCAF ≥5	p-value
Sex	31 (100.0)	Female	14 (56.0)	4 (66.7)	1.000 (a)
​	​	Male	11 (44.0)	2 (33.3)	​
Age	31 (100.0)	Mean (SD)	70.5 (10.9)	58.5 (17.0)	**0.038** (b)
Primary melanoma site	31 (100.0)	Cutaneous	23 (92.0)	4 (66.7)	0.159 (a)
​	​	Uveal	2 (8.0)	1 (16.7)	​
​	​	MUP	0 (0.0)	1 (16.7)	​
AJCC stage	31 (100.0)	IIB	1 (4.0)	0 (0.0)	1.000 (a)
​	​	III	9 (36.0)	2 (33.3)	​
​	​	IV	15 (60.0)	4 (66.7)	​
T	31 (100.0)	T0	0 (0.0)	1 (16.7)	0.581 (a)
​	​	T1	3 (12.0)	1 (16.7)	​
​	​	T2	3 (12.0)	0 (0.0)	​
​	​	T3	6 (24.0)	1 (16.7)	​
​	​	T4	8 (32.0)	2 (33.3)	​
​	​	Tx	5 (20.0)	1 (16.7)	​
N	31 (100.0)	N0	9 (36.0)	2 (33.3)	0.759 (a)
​	​	N1	7 (28.0)	3 (50.0)	​
​	​	N2	5 (20.0)	0 (0.0)	​
​	​	N3	4 (16.0)	1 (16.7)	​
M	31 (100.0)	M0	10 (40.0)	2 (33.3)	1.000 (a)
​	​	M1	15 (60.0)	4 (66.7)	​
Therapy	28 (90.3)*	Anti-CTLA-4 + anti-PD-1	12 (52.2)	2 (40.0)	1.000 (a)
​	​	Anti-PD-1	11 (47.8)	3 (60.0)	​
Progressive disease	31 (100.0)	No	16 (64.0)	3 (50.0)	0.653 (a)
​	​	Yes	9 (36.0)	3 (50.0)	​
Death	31 (100.0)	No	19 (76.0)	5 (83.3)	1.000 (a)
​	​	Yes	6 (24.0)	1 (16.7)	​

AJCC, american joint committee on cancer; T, tumor size (Tx, primary tumor cannot be assessed); N, lymph node positivity; M, distant metastasis (according to TNM, classification); MUP, melanoma of unknown primary; PD-1, programmed cell death protein 1; CTLA-4, cytotoxic T-lymphocyte-associated protein 4; SD, standard deviation. (a) Fisher’s exact test; (b) two-sided T-test. Significant p-values are highlighted in bold font.

* Three patients did not receive therapy.

Regarding laboratory data, no significant differences were observed when comparing the laboratory parameters of patient groups with low or high cCAF counts (Table 5).

**TABLE 5:** Laboratory characteristics of the patient cohort according to cCAF groups.

Variable	Total n	Level	cCAF <5	cCAF ≥5	p-value
LDH [U/L]	31	Median (IQR)	260 (164)	231 (108)	0.764 (a)
S100B [µg/L]	30	Median (IQR)	0.27 (1.16)	0.1 (0.28)	0.374 (a)
D-dimers [mg/L]	26	Median (IQR)	0.71 (2.08)	0.86 (2.12)	0.696 (a)
CRP [mg/L]	23	Median (IQR)	14.5 (23.5)	8 (35)	0.963 (a)
Neutrophil count [x10^9^/L]	31	Median (IQR)	5.76 (1.91)	4.86 (1.6)	0.516 (a)
Lymphocyte count [x10^9^/L]	31	Mean (SD)	1.54 (0.62)	1.56 (0.39)	0.916 (b)
NLR	31	Median (IQR)	3.55 (2)	2.8 (3.12)	0.575 (a)

LDH, lactate dehydrogenase; CRP, C-reactive protein; NLR, neutrophil/lymphocyte ratio; SD, standard deviation; IQR, interquartile range. (a) Mann–Whitney U test, (b) Student’s t-test.

### 3.3 Prognostic value of cCAFs in melanoma patients receiving ICI

**FIGURE 3:**
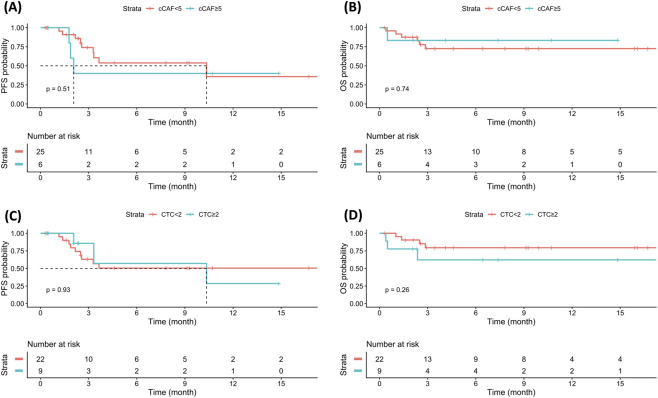
Kaplan-Meier survival curves for progression-free survival (PFS) and overall survival (OS) defined by cCAF and CTC numbers. Patients were divided into two groups based on their cCAF numbers: those with low cCAFs (<5 cCAFs) and those with high cCAFs (≥5 cCAFs). Survival curves based on the Kaplan-Meier method were created for the cCAF subgroups regarding PFS **(A)** and OS **(B)**, as well as the CTC subgroups (low CTCs (<2 CTCs) and high CTCs (≥2 CTCs)) for PFS **(C)** and OS **(D)**. Statistical significance in all four plots was determined using the log-rank test.

To evaluate the prognostic value of cCAF detection in the blood of melanoma patients, we analyzed the progression-free survival (PFS) and overall survival (OS) in our cohort. No significant differences were observed between patients with low cCAFs (<5) and high cCAFs (≥5), nor between those with low CTCs (<2) and high CTCs (≥2) (Figure 3). However, patients with a high number of cCAFs appear to have a trend toward shorter median progression-free survival of 2.07 months compared to 10.35 months in patients with low cCAF counts, although this difference is not statistically significant (p = 0.51) (Figure 3A).

As the stratification based on cCAFs or CTCs alone did not show statistically significant differences regarding PFS and OS, we analyzed the complementary value of LDH and S100B, which are used as established blood-based tumor markers in melanoma patients. First, we evaluated the prognostic effects of S100B and LDH alone in our cohort. Patients with high S100B (≥0.152 μg/L) or high LDH (≥245 U/L) had an impaired PFS time compared to those with low protein S100B (median PFS: 3.29 months vs. median PFS not reached, 95% CI: 2.17 – NA, p = 0.18) or LDH concentrations (median PFS: 3.29 months vs. median PFS not reached, 95% CI: 2.07 – NA, p = 0.25), although not statistically significant (Supplementary Figures S1A, S1B). With respect to OS, we observed a statistically significantly higher OS probability in patients with low S100B concentrations (p = 0.0094) and a strong trend of a longer OS in patients with non-elevated LDH levels (p = 0.071) (Supplementary Figures S1C, S1D).

**FIGURE 4:**
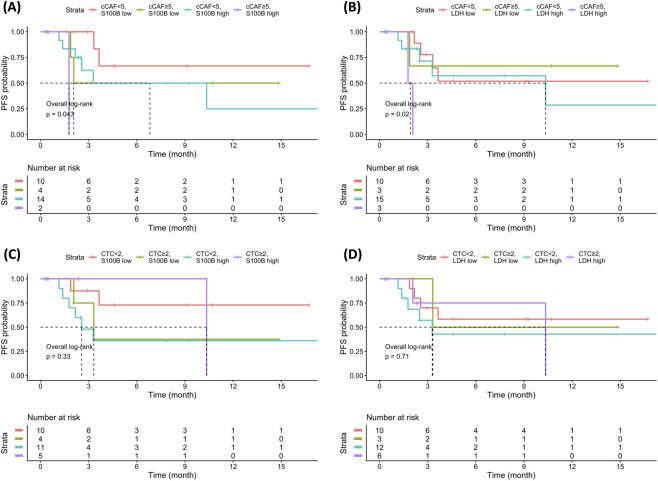
Kaplan-Meier survival curves for progression-free survival (PFS) defined by cCAF and CTC counts, combined with S100B or LDH. Progression-free survival curves based on the Kaplan-Meier method showing cCAF subgroups in combination with S100B **(A)** and LDH **(B)**. Patients were divided into four groups based on the number of detected cCAFs (low: <5/high: ≥5) and S100B (low: <0.152 μg/L/high: ≥0.152 μg/L) or LDH (low: <245 U/L/high: ≥245 U/L). Similarly, CTCs were divided into four groups based on the number of detected CTCs (low: <2, high: ≥2) and S100B or LDH. Kaplan-Meier plots showing PFS for the CTC subgroups in combination with S100B **(C)** and LDH **(D)** were plotted. Statistical significance was determined using the log-rank test, and the overall log-rank p-value is shown in the plots.

To determine whether the addition of the measured serum levels of S100B and LDH enhances prognostic accuracy, we combined them with the cCAF and CTC status for survival analysis (Figure 4). The cCAF status in combination with S100B or LDH led to significant differences in survival times (overall log-rank p = 0.043 and 0.02, respectively) (Figures 4A,B). Patients with low cCAF numbers and low S100B concentrations had the longest PFS time and did not reach the median PFS, whereas patients with elevated cCAFs and high S100B concentrations had a shorter PFS time with a median of 1.77 months. For patients with ≥5 cCAFs, but non-elevated S100B concentrations, the median PFS was 2.07 months (95% CI: 1.87 – NA), and for those with low cCAFs but high S100B, it was 6.82 months (95% CI: 2.56 – NA) (p = 0.850). The most notable difference in PFS time was observed between the low cCAFs/low S100B and high cCAFs/high S100B subgroups in the multiple comparison testing (p = 0.028) (Figure 4A).

With respect to LDH, patients with high cCAF numbers and high LDH concentrations progressed earlier, with a median PFS of 1.92 months (95% CI: 1.77 – NA). Patients with a combination of low cCAFs/low LDH and high cCAFs/low LDH did not reach median PFS, and patients with low cCAFs and high LDH had a median PFS of 10.35 months (95% CI: 2.5 – NA) (Figure 4B).

Furthermore, we investigated whether the combination of CTCs with established tumor markers in melanoma could enhance prognostic power. The combination of CTCs with S100B and LDH showed a trend of an increased risk stratification for PFS in patients with high CTCs and high S100B or high LDH levels, compared to the other combinations, but did not reach statistical significance (Figures 4C,D).

The further combination of CTCs and cCAFs with LDH or S100B did not lead to an increase in prognostic power for PFS in our cohort (Supplementary Figure S2).

## 4 Discussion

In this study, we have demonstrated that circulating CAFs can be detected in the blood of approximately half of the advanced melanoma patients in our cohort and are associated with a shorter, but not statistically significant, progression-free survival. Nevertheless, combining cCAFs with established blood-based markers such as S100B or LDH can support risk stratification and thus enhance prognostication in melanoma patients as shown in our exploratory analysis.

As previously mentioned, this is the first time cCAFs have been identified in individuals with melanoma to the best of our knowledge; therefore, no studies are available for direct comparison of our findings. Earlier studies in other solid tumor entities detected cCAFs in patients with breast, prostate, colorectal, gynecological, renal, cholangiocellular, lung, pancreatic, and gastrointestinal cancers (Jones et al., 2013; Ao et al., 2015; Ortiz-Otero et al., 2020; Jiang et al., 2021; Sharma et al., 2021; Muchlińska et al., 2023; Booijink et al., 2024; Götze et al., 2025). The positivity rate for cCAFs in these studies varied widely, ranging from 3.3% to 100%, with an average detection rate of 67% (Weber et al., 2025), which is consistent with our findings of cCAF detection in 50% of melanoma patients. The wide variation of CAF positivity rates is likely due to different methods used for enrichment and characterization in cCAFs, the high heterogeneity of the (c)CAFs, and the fact that no standardized method has been established to date (Weber et al., 2025). Furthermore, some studies have reported that cCAFs are present in a higher percentage of patients with distant metastasis than in those without metastasis or with only lymph node metastasis (Jones et al., 2013; Ao et al., 2015; Jiang et al., 2021; Muchlińska et al., 2023). However, we observed similar numbers of cCAF-positive patients in both groups of our cohort, comparable to a study by Sharma et al. (Sharma et al., 2021). These findings may reflect, in addition to methodological variability, also biological heterogeneity, but should be carefully interpreted and validated in further studies due to the low number of patients with locally (advanced) tumors in our cohort.

An advantage of our study is the use of a fully automated and standardized cCAF isolation method based on depletion rather than enrichment. This approach reduces selection bias and allows for the capture of a broader and more representative range of circulating cells. However, the staining panel employed in this study included only α-SMA as a single marker for cCAFs detection, which may not fully capture the heterogeneity of circulating fibroblasts. The addition of other cCAF markers, such as fibroblast activation protein (Jiang et al., 2021; Sharma et al., 2021; Booijink et al., 2024; Götze et al., 2025), could facilitate the detection of additional cCAFs that lack α-SMA expression and consequently influence detection rates. This would also allow for the distinction between different cCAF subtypes, as shown in previous studies for other tumors (Muchlińska et al., 2023; Götze et al., 2025; Woo et al., 2025). Currently, no marker panel for cCAFs in melanoma has been established. However, CAF subtypes utilizing different markers have been identified in melanoma tissue samples (Papaccio et al., 2021; Forsthuber et al., 2024; Lujano Olazaba et al., 2024), indicating that certain CAF subtypes may have different functions in tumor progression.

In our cohort, patients with elevated cCAF counts had shorter progression-free survival under ICI therapy, although this result was not statistically significant. A reason for this could be CAF heterogeneity, as some CAF subgroups are associated with poorer prognosis due to lower response to therapy, likely because of a dense extracellular matrix (Forsthuber et al., 2024). The correlation between cCAF levels and worse prognosis, as well as lower probability of survival, was demonstrated by Ortiz-Otero et al. across multiple tumors in metastatic patients (Ortiz-Otero et al., 2020). Götze et al. detected a lower median OS of 3.2 months in metastatic PDAC patients with more than 15 cCAFs compared to 14.2 months in patients with fewer cCAFs (Götze et al., 2025). These findings further suggest that cCAFs represent a clinically meaningful liquid biopsy analyte.

Surprisingly, cCAFs showed no significant associations with clinico-pathological parameters other than patient age, which may reflect age-dependent differences in fibroblast biology, as higher stromal scores in younger individuals have been demonstrated in melanoma patients (Ning et al., 2021). Previous studies in other cancer entities showed only a correlation of increased cCAF numbers and distant or lymph node metastasis, as mentioned above, but did not state a correlation with further clinico-pathological variables (Ortiz-Otero et al., 2020). However, investigations on healthy donors from other research groups consistently demonstrate that cCAFs are absent in non-cancer individuals, supporting their tumor specificity (Jones et al., 2013; Ao et al., 2015; Ortiz-Otero et al., 2020; Jiang et al., 2021; Sharma et al., 2021; Muchlińska et al., 2023; Booijink et al., 2024). Therefore, cCAFs may be partly tumor-independent, reflecting processes not associated with standard clinical variables and giving further insights into the biology of the tumor.

Although CTC status did not correlate with overall survival in our cohort, higher CTC counts were associated with elevated CRP and D-dimer levels in line with current literature that may indicate increased tumor cell dissemination in melanoma patients (Mego et al., 2015; Desch et al., 2017; Kött et al., 2024).

A limitation of our study is the relatively small cohort size, which limits the statistical power and the generalizability of our findings. Moreover, the here presented cut-off values were determined using maximally selected rank statistics in a moderately sized cohort of melanoma patients, which should be interpreted with caution and considered exploratory. To validate and expand these observations, future studies should involve larger patient populations. In this context, a multicenter study would better capture the heterogeneity of patient cohorts and enhance the statistical robustness of the prognostic power and validity of the cut-offs chosen. Another important question would be to evaluate the relevance of the co-detection of cCAFs and CTCs, as breast cancer patients positive for both showed a significantly higher frequency of metastases compared to patients positive for only one marker (Muchlińska et al., 2023). This was unfortunately not possible in our cohort, as the sample size for subgroup analysis was limited. Additionally, analyzing longitudinal samples would be valuable to assess changes in cCAFs over time during ICI therapy. Furthermore, functional studies of cCAFs are necessary to better understand their role in circulation and their potential influence on disease progression and treatment response.

**FIGURE 5:**
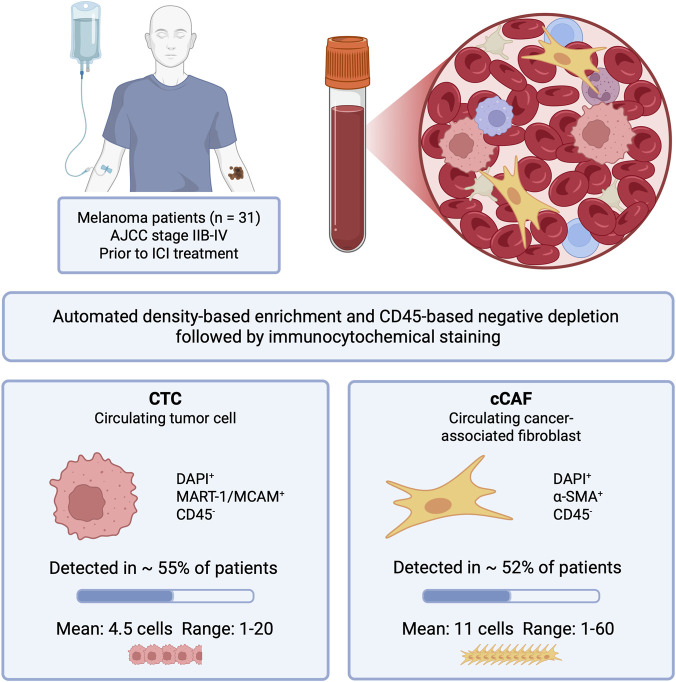
Co-detection of cCAFs and CTCs in melanoma patients. Whole blood samples from 31 melanoma patients (stage IIB-IV) were enriched using automated density-based enrichment with CD45-negative depletion and stained with DAPI, MART-1, MCAM, α-SMA, and CD45. CTCs were detected in 54.8% of patients with a mean of 4.5 cells, while cCAFs were detected in approximately 52.6% of patients with a higher mean of 11 cells. Created in BioRender. Reese, K. (2026) https://BioRender.com/4jtug0h.

In conclusion, our study shows for the first time that circulating cCAFs can be detected in melanoma patients. We successfully used a method for co-detecting CTCs and cCAFs from whole blood samples (Figure 5). CTCs and cCAFs were found in a similar number of patients, but cCAFs appeared in higher mean numbers compared to CTCs. Since higher cCAF levels showed a trend toward shorter PFS in our cohort, cCAFs should be tested in future larger studies as a potential biomarker to predict progression risk in melanoma patients. In addition, the detection of cCAFs opens a new avenue for their functional characterization in further studies.

## Data Availability

The original contributions presented in the study are included in the article/Supplementary Material, further inquiries can be directed to the corresponding author.
