# Supplementary material for: Detection and prognostic role of circulating cancer-associated fibroblasts in the blood of melanoma patients
Source: Front Cell Dev Biol. 2026 Jun 1;14:1774206. doi: 10.3389/fcell.2026.1774206 (PMC13265522; doi:10.3389/fcell.2026.1774206)
Supplement: Supplementary file 1 [file DataSheet1.docx]

Supplementary Material

Supplemental Figure 1. Kaplan-Meier survival curves for progression-free survival (PFS) and overall survival (OS) defined by S100B and LDH levels. Patients were subgrouped by their baseline S100B and LDH levels into the following groups: S100B low (<0.152 µg/L), S100B high (≥0.152 µg/L), LDH low (<245 U/L), and LDH high (≥245 U/L). Survival curves based on the Kaplan-Meier method were created for PFS in the S100B (A) and LDH (B) subgroups, and for OS in the S100B (C) and LDH (D) subgroups. Statistical significance was determined using the log-rank test.

Supplemental Figure 2. Kaplan-Meier survival curves for progression-free survival (PFS) defined by CTC and cCAF numbers in combination with S100B or LDH levels. Patients were divided into groups based on their low or high CTC (low: <2 / high: ≥2) and cCAF (low: <5 / high: ≥5) counts combined with S100B (low: <0.152 µg/L / high: ≥0.152 µg/L) (A) or LDH (low: <245 U/L / high: ≥245 U/L) (B) concentrations. Statistical significance was determined using the overall log-rank test.
